# Associations between attention-deficit/hyperactivity disorder and autoimmune diseases are modified by sex: a population-based cross-sectional study

**DOI:** 10.1007/s00787-017-1056-1

**Published:** 2017-10-05

**Authors:** Tor-Arne Hegvik, Johanne Telnes Instanes, Jan Haavik, Kari Klungsøyr, Anders Engeland

**Affiliations:** 1 Department of Biomedicine, University of Bergen, Jonas Lies vei 91, N-5009 Bergen, Norway; 2 K.G. Jebsen Centre for Neuropsychiatric Disorders, University of Bergen, Jonas Lies vei 91, N-5009 Bergen, Norway; 3 Department of Global Public Health and Primary Care, University of Bergen, Bergen, Norway; 4 Division of Psychiatry, Haukeland University Hospital, Bergen, Norway; 5 Domain for Health Data and Digitalization, Norwegian Institute of Public Health, Bergen, Norway; 6 Department of Pharmacoepidemiology, Norwegian Institute of Public Health, Bergen/Oslo, Norway

**Keywords:** ADHD, Autoimmunity, Neuropsychiatry, Comorbidity, Psoriasis, Neuroimmunology

## Abstract

Several studies have demonstrated associations between neuropsychiatric disorders, such as attention-deficit/hyperactivity disorder (ADHD), and the immune system, including autoimmune diseases. Since ADHD and many autoimmune diseases show sex-specific properties, such associations may also differ by sex. Using Norwegian national registries, we performed a cross-sectional study based on a cohort of 2,500,118 individuals to investigate whether ADHD is associated with common autoimmune diseases. Associations between ADHD and autoimmune diseases in females and males were investigated with logistic regression and effect modification by sex was evaluated. Several subanalyses were performed. The strongest association was found between ADHD and psoriasis in females, adjusted odds ratio (adjOR) = 1.57 (95% confidence interval: 1.46–1.68) and males, adjOR = 1.31 (1.23–1.40); *p* value for interaction < 0.0001. Furthermore, among females, ADHD was associated with Crohn’s disease, adjOR = 1.44 (1.16–1.79) and ulcerative colitis, adjOR = 1.28 (1.06–1.54). In contrast, males with ADHD had lower odds of Crohn’s disease, adjOR = 0.71 (0.54–0.92), in addition to a trend for lower odds of ulcerative colitis, adjOR = 0.86 (0.71–1.03); *p* values for interaction < 0.0001 and 0.0023, respectively. In a group of females where information on smoking and body mass index was available, adjustment for these potential mediators did not substantially alter the associations. Our findings support previously reported associations between ADHD and diseases of the immune system. The associations differ by sex, suggesting that sex-specific immune-mediated neurodevelopmental processes may be involved in the etiology of ADHD.

**Electronic supplementary material:**

The online version of this article (doi:10.1007/s00787-017-1056-1) contains supplementary material, which is available to authorized users.

## Introduction

Attention-deficit/hyperactivity disorder (ADHD) is a neurodevelopmental disorder characterized by the symptoms of inattention, hyperactivity and impulsivity. The symptoms of this childhood onset condition often persist into adulthood [1]. Furthermore, patients often suffer from comorbid psychiatric disorders [2, 3] and face socioeconomic hardship [4]. The etiology of ADHD is largely unknown, but in twin studies, the heritability of the disorder has been estimated to be 70–80%, implicating a strong genetic basis [1, 5, 6]. Environmental factors and perinatal factors such as preterm birth and growth restriction have also been shown to influence the development of ADHD [1, 7, 8].

Numerous studies have reported associations between neuropsychiatric disorders and immune system abnormalities [9–16]. However, these associations remain uncertain [17–19]. Likewise, several immune-related disorders, such as atopic dermatitis, asthma, ankylosing spondylitis, ulcerative colitis (UC), juvenile arthritis, autoimmune thyroid disease and celiac disease have been associated with ADHD [20–23]. Additionally, maternal autoimmunity has been associated with offspring ADHD, implying that maternal immune system dysfunction may affect the in utero environment and again fetal neurodevelopment [21, 24]. Despite the genetic architecture of ADHD being relatively unknown, some tentative genetic associations between ADHD and the immune system have been noted. For example, a study on genetic pathways of ADHD, which was based on genome-wide association studies (GWAS), found an increased burden of polymorphisms in and around genes involved in toll-like receptor signaling [25]. These signaling pathways are highly involved in the innate immune responses and have also been shown to regulate hippocampal plasticity and neurogenesis, and memory formation [26]. Furthermore, the single nucleotide polymorphism (SNP) which showed the strongest association signal in a recent ADHD GWAS, which included more than 20,000 ADHD patients and 35,000 controls, is located in the gene *ST3GAL3* [27]. Knockout of the *ST3GAL3* gene affects both eosinophilic immune responses [28] and brain development [29].

ADHD has an approximate male:female ratio of 3:1 during childhood and adolescence, which approaches 1:1 in adults [1]. Moreover, ADHD displays sex-specific manifestations [30]. For example, females are more often primarily affected by inattention, whereas males more often display additional symptoms of hyperactivity and impulsivity [31]. Likewise, autoimmune diseases have prevalence rates and symptom burdens that may differ by sex [32–34]. Interestingly, GWASs have reported SNPs to be associated with an autoimmune disease in one sex, but not the other [35] and genetic effects being in opposite directions depending on sex have been reported [36, 37]. Further, sex hormones are believed to have immune-modulating properties, as exemplified by symptom remission of multiple sclerosis and rheumatoid arthritis during pregnancy [33, 34]. Neural functioning might also be regulated by these hormones, as demonstrated by menstrual cycle-associated seizures of certain types of epilepsy [38]. Besides, behavior may be affected by sex hormones. For instance, females exposed to elevated prenatal androgen levels may develop more aggressive behavior later in life as compared to non-exposed females [39], and moreover, aggressive behavior is associated with ADHD [40].

In sum, if sex-specific genetic pleiotropy, or other sex-specific mechanisms, underlie any associations between ADHD and autoimmunity, these associations may differ substantially by sex. In other words, sex could be an effect measure modifier.

To further explore possible associations between autoimmunity and ADHD, and to evaluate whether these associations vary by sex, we conducted a large cross-sectional study based on Norwegian national registries.

## Materials and methods

### The Medical Birth Registry of Norway (MBRN)

The Medical Birth Registry of Norway (MBRN) was established in 1967 to collect medical and familial information on parents and births in Norway [41]. Registration in the MBRN is mandatory for all pregnancies from 16 completed weeks of gestation, and is based on a standardized notification form. Maternal smoking habits have been included in the registry since December 1998, but is one of few variables where mothers can refuse registration. Still, for approximately 84% of the births smoking information is registered. Since 2006, electronic notification of births to the MBRN has been introduced gradually, based on standardized extraction from medical records at the delivery units, and has included information on maternal height and weight before and at the end of pregnancy. However, it was not until 2014 that electronic notification was in place at all delivery units and in 2013, information on height and weight was still missing for approximately 36% of the pregnancies.

Data for the current study was obtained for all live births in the MBRN from January 1st 1967 to December 31st 2013.

### The Norwegian Prescription Database (NorPD)

The Norwegian Prescription Database (NorPD) was established in 2004 and provides information on all medical prescriptions dispensed to patients from all Norwegian pharmacies, and includes the Anatomical Therapeutic Chemical Classification System (ATC) codes [42]. Information on medication received during hospitalization is not available on an individual basis. From 2008, the NorPD has included information on diagnostic codes for reimbursed medication based on either the International Classification of Primary Care (ICPC) or the International Statistical Classification of Diseases and Related Health Problems 10th version (ICD-10), used in specialist health care. From 2004 to 2008, the NorPD also included diagnostic codes for prescribed reimbursed medication. However, these diagnostic codes were less specific, and therefore not used in this study.

For the present study, information was obtained for all dispensed drugs between January 1st 2004 and December 31st 2015.

### The National Education Database

The National Education Database holds information on the education of all Norwegian citizens from the age of 16 years. The database covers all levels of education from primary school to PhD-level. For the present study, data on education as registered in 2012 was available.

### The National Registry

The National Registry supplied information on emigration and dates of death.

### Included individuals and record linkage

All individuals registered in the MBRN as born between 1967 and 2011, who were alive and residing in Norway on December 31st 2015, were included in the study. In addition, the mothers of those registered in the MBRN between 1998 and 2013, were identified for supplementary analyses allowing adjustment for body mass index (BMI) and smoking. Mothers who had died or emigrated by December 31st 2015 were excluded from these supplementary analyses (see below).

All Norwegian citizens have a unique personal identification number. This number was used to establish linkage between the registries.

### ADHD case definition

ADHD cases were defined as all individuals, regardless of age, who had been dispensed reimbursed ADHD medication (ATC N06BA) (*n* = 63,721), without reimbursement codes for “narcolepsy”, G47 in ICD-10 and “sleep disturbance”, P06 in ICPC (*n* = 407), during 2004–2015.

The remaining population served as the comparison group (*n* = 2,436,397).

### Autoimmune diseases

Autoimmune disease cases were defined from reimbursement codes or specific dispensed drugs corresponding to one of several predefined and common autoimmune diseases. The set of diseases was based on a Danish study describing the prevalence of 30 autoimmune diseases [43].

The estimated coverage of the autoimmune disease cases was compared with the reported prevalence rates of the autoimmune diseases in the general population by utilizing Eaton et al. 2010 [43] in addition to Norwegian and Swedish prevalence studies. Autoimmune diseases where the available reimbursement codes were considered too unspecific, that had unlikely prevalence estimates, or with less than 1000 cases in total (< 4 pr 10,000), were excluded. Nine autoimmune diseases passed the inclusion criteria (see Table 1) and were included in the study.

**Table 1:** Definitions of ADHD and the autoimmune diseases assessed in the primary analyses

Disease/disorder	Definition of case
ADHD	Prescribed and dispensed at least one reimbursed drug once with ATC-code N06BA excluding those with reimbursement code ICD-10 G47 (narcolepsy) or ICPC P06 (sleep disturbance)
Ankylosing spondylitis	Prescribed and dispensed at least one drug once with reimbursement code ICD-10 M45
Crohn’s disease	Prescribed and dispensed at least one drug once with reimbursement code ICD-10 K50
Iridocyclitis	Prescribed and dispensed at least one drug once with reimbursement code ICD-10 H20
Multiple sclerosis^a^	Prescribed and dispensed at least one drug once with ATC-code L03AB07, L03AB08, L03AB13, L03AX13, L04AA23, L04AA27, L04AA31, L04AA34, L04AC01, N07XX07 or N07XX09
Psoriasis	Prescribed and dispensed at least one drug once with reimbursement code ICD-10 L40 or ICPC S91
Rheumatoid arthritis	Prescribed and dispensed at least one drug once with reimbursement codes ICD-10 M05 or M06
SLE	Prescribed and dispensed at least one drug once with reimbursement code ICD-10 M32
Type 1 diabetes	Prescribed and dispensed at least one drug once with reimbursement code ICD-10 E10 or ICPC T89, excluding those who have been dispensed at least one drug once with ATC-code A10B
Ulcerative colitis	Prescribed and dispensed at least one drug once with reimbursement code ICD-10 K51

*ADHD* attention-deficit/hyperactivity disorder, *ATC* Anatomical Therapeutic Chemical Classification System, *ICD-10* International Statistical Classification of Diseases and Related Health Problems 10, *ICPC* International Classification of Primary Care, *SLE* systemic lupus erythematosus

^a^ The ICD-10 and ICPC codes for multiple sclerosis are not used in Norway at drug prescribement due to health-regulatory reasons. ATC codes for multiple sclerosis-specific drugs therefore defined multiple sclerosis

### Statistical analysis

Possible associations between ADHD and the autoimmune diseases were estimated as odds ratios (OR) with 95% confidence intervals (CI) using logistic regression. *p* values are presented uncorrected for multiple testing. The threshold for statistical significance was adjusted *ad modum* Bonferroni (*p* = 0.05 divided by the number of autoimmune diseases included in the primary analyses) to *p* = 0.0056. The threshold for nominal significance was defined as *p* = 0.05. Data management and statistical analyses were performed with R [44], RStudio [45] and IBM SPSS [46].

### Primary analyses

In the primary analyses, associations between autoimmune diseases and ADHD were investigated with adjustment for age as a continuous covariate, except for type 1 diabetes where age was categorized into four (years of age in 2015: 4–10; 11–15; 16–20; 21–48). All analyses were stratified by sex [1, 30–34]. Effect modification by sex was evaluated on a multiplicative scale including an interaction term in the logistic regression model, and statistical significance was evaluated by Wald test.

Socioeconomic status as defined by maternal education was adjusted for as a categorical covariate with three categories, low (< 10 years of education), medium (10–12 years) and high (> 12 years).

Statistically significant associations in the primary analyses were further investigated in supplementary analyses concerning potential confounders, mediators and biases.

### Adjustment for smoking and body mass index (mother analyses)

Tobacco smoking and BMI may be mediating factors between ADHD and autoimmune diseases. Smoking is known to be associated with ADHD [47, 48] and has been associated with increased risk of several autoimmune diseases in prospective studies [49–51]. The similar applies to BMI in ADHD [20, 52] and autoimmunity [53–56]. To conduct a sensitivity analysis on whether the associations discovered in the main analyses were mediated mainly through smoking and/or BMI, a new study population including data on smoking and BMI was defined. The MBRN supplied data on smoking for women giving birth from December 1998 to 2013, and these mothers defined the study population when assessing the effect of smoking (from now on referred to as the “mother analyses”). Smoking during pregnancy was used as a proxy for smoking at linkage. As proxy for BMI at linkage, pre-pregnant BMI (kg/m^2^) of the mothers was used. Mothers with registered height below 130 cm or BMI below 15 or above 60 were set to missing as these values were considered biologically implausible. Socioeconomic status was defined as the education of the mother in 2012 categorized into three: low (< 10 years), medium (10–12 years) and high (> 12 years). For females who had given birth to several children, only data from the last registered birth was included.

Logistic regression was used to investigate associations between ADHD and autoimmune diseases among these mothers with and without adjustment for the mother’s smoking habits and with education as covariate. Further, a similar logistic regression was conducted with the inclusion of BMI, modelled as a continuous covariate, in addition to smoking and education. Substantial attenuation of the estimated associations between ADHD and autoimmune diseases when adjusting for smoking and BMI, would indicate that much of the effect of ADHD on these diseases might be mediated through these mediators [57, 58].

Several additional subanalyses were also conducted, when possible, to scrutinize statistically significant associations identified in the primary analyses (see supplemental material).

## Results

### Demographics

We identified a total of 2,500,118 individuals in the MBRN fulfilling our inclusion criteria for the primary analyses, 1,219,669 females and 1,280,449 males.

22,878 (1.9%) of the females had ADHD with the highest prevalence among those born in 1993 (3.5%). Of the males, 40,843 (3.2%) had ADHD, with the highest prevalence among those born in 1996 (6.8%) (supplementary Fig. 1). ADHD was associated with lower socioeconomic status, as defined by maternal educational level (see Table 2).

**Table 2:** Characteristics of the study population in the primary analyses

Disease/disorder	*n* (per 10 000)	Mean age in 2015	Females (%)	Maternal education %			
				Low (< 10 years)	Medium (10–12 years)	High (> 12 years)	Information missing
Total study sample	2,500,118	25.8	1,219,669 (48.8)	23.1	42.0	33.9	0.1
ADHD	63,721 (255)	23.4	22,878 (35.9)	31.5	42.1	25.7	0.7
Ankylosing spondylitis	3504 (14)	37.4	1480 (42.2)	28.9	47.6	23.0	0.5
Crohn’s disease	6292 (25)	32.1	3284 (52.2)	27.6	46.0	25.9	0.5
Iridocyclitis	7596 (30)	34.0	3470 (45.7)	26.3	46.7	26.6	0.4
Multiple sclerosis	3739 (15)	38.1	2621 (70.1)	29.6	49.6	20.5	0.4
Psoriasis	62,418 (250)	33.8	32,190 (51.6)	29.6	46.2	23.7	0.6
Rheumatoid arthritis	8560 (34)	37.2	5662 (66.1)	30.9	47.8	20.8	0.4
SLE	1197 (5)	35.9	1024 (85.5)	30.2	45.6	23.6	0.7
Type 1 diabetes	14,273 (57)	29.5	6041 (42.3)	23.7	46.4	29.6	0.4
Ulcerative colitis	10,960 (44)	34.3	5392 (49.2)	26.3	47.3	26.0	0.4

*ADHD* attention-deficit/hyperactivity disorder, *SLE* systemic lupus erythematosus

The total number of patients per autoimmune disease ranged from 1197 (5 per 10,000) for systemic lupus erythematosus (SLE) to 62,418 (250 per 10,000) for psoriasis. The female-to-male ratios varied across the autoimmune diseases, with 42.3% of type 1 diabetes patients being female, to 85.5% of SLE patients. All autoimmune diseases increased in prevalence with age (supplementary Fig. 1). All autoimmune diseases were associated with lower socioeconomic status.

**Fig. 1:**
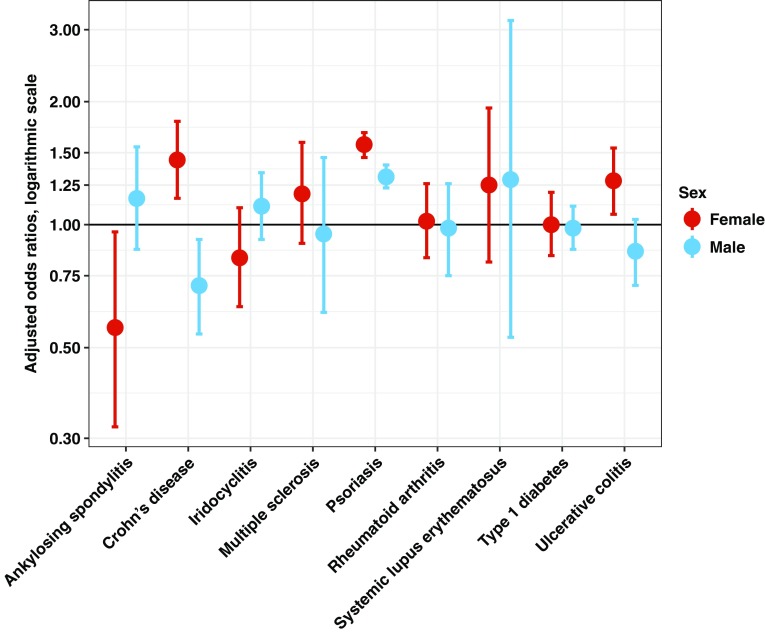
Sex-specific associations (odds ratios with 95% confidence intervals adjusted for age and maternal education) between ADHD and the autoimmune diseases investigated in the primary analyses

### Primary analyses

ADHD was significantly associated with increased odds of psoriasis in both females, adjusted (adj) OR = 1.57 (95% CI 1.46–1.68) and males, adjOR = 1.31 (95% CI 1.23–1.40). Associations were significantly stronger for females than males, *p* value for interaction by sex = 4.4 × 10^−6^.

Sex differences were even larger for Crohn’s disease (CD) and UC: Females with ADHD had a significantly higher odds of CD, adjOR 1.44 (95% CI 1.16–1.79), and UC, adjOR = 1.28 (95% CI 1.06–1.54), than females without ADHD. Males with ADHD, on the other hand, seemed protected, with a lower odds of CD than males without ADHD, adjOR = 0.71 (95% CI 0.54–0.92; nominal statistically significant), and a tendency to lower odds of UC, adjOR = 0.86 (95% CI 0.71–1.03). There were significant interaction effects between ADHD and sex on the odds for both CD, *p* = 3.6 × 10^−5^, and UC, *p* = 0.0023. Despite not reaching the threshold for statistical significance, UC was taken to supplementary analyses as UC shares many characteristics with CD and displayed statistically significant interaction effects by sex.

ADHD was further associated with lower odds of ankylosing spondylitis among females, but only at nominal statistical significance, adjOR = 0.56 (95% CI 0.32–0.96). No association was found for males, adjOR = 1.16 (95% CI 0.87–1.55). A nominally significant interaction effect between ADHD and sex was noted, *p* = 0.021.

The primary analyses were also adjusted for prematurity, gestational age ≥ 37 weeks or < 37 weeks, with little effect on the results (data and results not presented).

See Table 3 for detailed results and Fig. 1 for graphical representation of the sex-specific associations between ADHD and the autoimmune diseases.

**Table 3:** Associations between ADHD and autoimmune diseases among males and females, and the p-value for the interaction between ADHD and sex, with adjustment for age and maternal education

Autoimmune disease	Females		Males		All
	Adjusted for age *n* = 1,219,669 ADHD *n* = 22,878	Adjusted for age and maternal education *n* = 1,207,694 ADHD *n* = 22,741	Adjusted for age *n* = 1,280,449 ADHD *n* = 40,843	Adjusted for age and maternal education *n* = 1,267,647 ADHD *n* = 40,544	*p* value of interaction between ADHD and sex adjusted for age and maternal education *n* = 2,475,341 ADHD n = 63,285
	OR (95% CI)	OR (95% CI)	OR (95% CI)	OR (95% CI)	*p*
Ankylosing spondylitis	*0.56 (0.32–0.96)*	*0.56 (0.32–0.96)*	1.17 (0.88–1.56)	1.16 (0.87–1.55)	*0.021*
Crohn’s disease	***1.47 (1.18–1.82)***	***1.44 (1.16–1.79)***	*0.71 (0.54–0.92)*	*0.71 (0.54–0.92)*	***3.6*** ***×*** ***10*** ^***-5***^
Iridocyclitis	0.84 (0.64–1.12)	0.83 (0.63–1.10)	1.11 (0.92–1.34)	1.11 (0.92–1.34)	0.084
Multiple sclerosis	1.20 (0.90–1.60)	1.19 (0.90–1.59)	0.95 (0.61–1.46)	0.95 (0.61–1.46)	0.35
Psoriasis	**1.60 (1.49–1.72)**	**1.57 (1.46–1.68)**	***1.34 (1.25–1.43)***	***1.31 (1.23–1.40)***	***4.4*** ***×*** ***10*** ^***-6***^
Rheumatoid arthritis	1.05 (0.85–1.28)	1.02 (0.83–1.26)	1.01 (0.78–1.30)	0.98 (0.75–1.26)	0.65
SLE	1.26 (0.82–1.94)	1.25 (0.81–1.93)	1.28 (0.52–3.13)	1.29 (0.53–3.16)	0.98
Type 1 diabetes^a^	1.00 (0.83–1.19)	1.00 (0.84–1.20)	0.98 (0.87–1.11)	0.98 (0.87–1.11)	0.79
Ulcerative colitis	*1.27 (1.06–1.53)*	*1.28 (1.06–1.54)*	0.86 (0.71–1.03)	0.86 (0.71–1.03)	***0.0023***

Italics: *p* < 0.05

Bold and italics: *p* < 0.0056

*ADHD* attention-deficit/hyperactivity disorder, *CI* confidence interval, *OR* odds ratio, *SLE* systemic lupus erythematosus

^a^ Age was categorized into four, years of age in 2015: 4–10; 11–15; 16–20; 21–48 and adjusted for as a nominal covariate

### Adjustment for smoking and BMI (mother analyses)

512,957 females gave their last birth between December 1998 and December 31st 2013 during which time smoking habits were registered in the MBRN. Of these, 373,672 (72.8%) were themselves also registered in the MBRN at their own birth. There was information on educational level for 497,005 (96.9%) of the mothers, and of these, information on smoking for 420,050 (84.5%). Of these 420,050, 73,891 (17.6%) were defined as smokers, and additional information on pre-pregnant BMI for was available for 110,008 (26.2%). The mean and standard deviation of pre-pregnant BMI was 24.4 and 4.8, respectively, and 13,304 (12.1%) of these 110,008 females were defined as smokers. Thus, data on educational level, smoking and BMI was available for 21.4% of all females delivering their last recorded birth since the introduction of smoking information in December 1998 and up to December 31st 2013. See supplementary Fig. 2 for flowchart.

In the mother analyses, ADHD was associated with increased odds of psoriasis, adjOR = 1.62 (95% CI 1.44–1.81) also after additional adjustment for smoking, adjOR = 1.49 (95% CI 1.33–1.67) and BMI, adjOR = 1.29 (95% CI 1.04–1.60).

ADHD was associated with CD, adjOR = 1.77 (95% CI 1.23–2.54) and UC, adjOR = 1.87 (95% CI 1.42–2.46). Adjustment for smoking did not materially change ADHD’s association with CD, adjOR = 1.63 (95% CI 1.13–2.34) nor UC, adjOR = 1.90 (95% CI 1.45–2.50). Similarly, additional adjustment for BMI did not alter the ADHD-CD association, adjOR = 2.20 (95% CI 1.24–3.88) nor the ADHD-UC association, adjOR = 2.10 (95% CI 1.30–3.39).

Similar analyses stratified by smoking and overweight, BMI < 25 or ≥ 25, were also conducted. The results were in line with the presented findings (data and results not presented). See Table 4 for detailed results.

**Table 4:** Associations between ADHD and Crohn’s disease, ulcerative colitis and psoriasis among females with adjustment for education, smoking and body mass index

Females	Adjusted for education^a^ *n* = 420,050 ADHD *n* = 5636		Adjusted for education and smoking *n* = 420,050 ADHD *n* = 5636	Adjusted for education and smoking^b^ *n* = 110,008 ADHD *n* = 1814		Adjusted for education, smoking and BMI *n* = 110,008 ADHD *n* = 1814
	*n*	OR (95% CI)	OR (95% CI)	*n*	OR (95% CI)	OR (95% CI)
Crohn’s disease	1225	1.77 (1.23–2.54)	1.63 (1.13–2.34)	329	2.27 (1.29–4.01)	2.20 (1.24–3.88)
Psoriasis	14,226	1.62 (1.44–1.81)	1.49 (1.33–1.67)	3919	1.39 (1.12–1.72)	1.29 (1.04–1.60)
Ulcerative colitis	2479	1.87 (1.42–2.46)	1.90 (1.45–2.50)	676	2.00 (1.24–3.22)	2.10 (1.30–3.39)

*ADHD* attention-deficit/hyperactivity disorder, *BMI* body mass index, *CI* confidence interval, *OR* odds ratio

^a^ Restricted to females with information on smoking

^b^ Restricted to females with information on BMI

### Psoriasis, Crohn’s disease and ulcerative colitis

As a robust positive association between psoriasis and ADHD was identified, this association was further examined, both in regards to psoriasis case definition and age- and period effects. The supplementary analyses confirmed the results of the primary analyses (see supplementary material for both specification of analyses and results).

The diagnoses CD and UC partly overlapped as 2334 individuals in the primary analyses were defined as having both conditions (37.1% of the CD patients and 21.3% of the UC patients). Supplementary analyses were conducted after redefining all individuals with both CD and UC as having neither. The results confirmed the positive associations in females, and the interaction by sex, but the negative associations in males were now not present (see supplementary material for both specification of analyses and results). To assess age- and period effects of ADHD on CD and UC, analyses stratified on birth years, 1967–1985 and 1986–2011, were conducted. For CD, the results were in line with the main analyses for both individuals born 1967–1985 and those born 1986–2011, including the sex-effects. However, for UC, the positive association in females, and negative association in males, were only noted in those born 1967–1985. For those born 1986–2011, no associations were noted (see supplementary material for further specification of analyses and results).

## Discussion

In our large cross-sectional study based on population-wide registries, ADHD was clearly and positively associated with psoriasis. This association was present regardless of sex, but with a significantly stronger association in females than males. Furthermore, in females, ADHD was positively associated with CD and UC. In contrast, among males, ADHD showed a negative association with CD, and a similar tendency with UC.

Psoriasis is a skin disorder characterized by red scaly skin plaques, papules or patches and is generally considered an autoimmune disease [43]. The etiology behind psoriasis is complex, including environmental and lifestyle factors [50, 53, 59] and several genetic risk variants have been identified, mainly in and around genes involved in the immune response and skin barrier regulation [60]. In agreement with our findings, a Danish registry-based study noted a possible association between psoriasis and ADHD [21]. However, the association was not statistically significant (*p* = 0.09), which may be due to the study’s prospective study design where the autoimmune diseases had to debut prior to ADHD. In contrast, we utilized a cross-sectional design. Moreover, we investigated both children and adults, 4–48 years of age at linkage, whereas the Danish study only examined children and young adults, 5–22 years old at linkage, which lead to a smaller study sample. In addition, many of the individuals in the Danish study were simply too young to have developed psoriasis [59] or to have been diagnosed with ADHD [1].

Several different mechanisms may account for the association between ADHD and psoriasis. A recent family-based epidemiological study reported a significant genetic correlation between ADHD and psoriasis [61], indicating that there could be pleiotropic genetic effects in shared risk pathways. For example, complement factor C3 is highly expressed in both psoriatic lesions [62] and is important for synaptic pruning in the brain [63]. Lifestyle and environmental factors associated with ADHD, such as smoking and high BMI [20, 47, 48, 52], may also provoke psoriasis [50, 53]. However, in our mother analyses, adjustment for these risk factors did not attenuate the association, implying alternative etiological pathways [57, 58]. Furthermore, emotional and social stressors associated with ADHD [4, 64] could perhaps trigger psoriasis in predisposed individuals [65].

CD and UC are both diseases primarily affecting the gastrointestinal system [51]. They are considered separate disease entities, but share many similarities, both clinically and etiologically, and are referred to collectively as inflammatory bowel disease (IBD). More than 150 genetic risk variants have been identified for both, many of which are shared, and environmental factors are highly implicated in the etiology [51, 66, 67]. In a study from Taiwan, ADHD was associated with UC, but not CD [22]. However, the authors did not report sex-specific effects, raising the possibility that the common estimate may be biased, and that the sex-specific effects present in our study, were not identified. Moreover, the prevalence-ratio of CD to UC was > 10:1 among the controls, indicating possible age-effects in addition to ethnic differences.

The increased odds of CD and UC in females with ADHD, with a reverse relation in males is striking. In addition, ADHD females had significantly higher odds of psoriasis than ADHD males. Sex, including both hormonal and non-hormonal influences, is a key determinant of immune system functioning [33, 34], brain development, neural functioning and psychiatric disease [30, 31, 38, 39, 68, 69]. A possible etiology for the sex-specific effects may involve glial cells, which are neuron-and homeostasis-supportive cells of the nervous system with immunomodulatory properties [33, 68, 70]. Glial cells have been shown to modulate sex-determined neurodevelopmental processes, including synaptic patterning and neurite pruning [68, 69]. Further, there are studies suggesting a role for glial cells located along the gut in the etiology of CD and UC, in addition to several other gastrointestinal disorders [70].

Genetically, pleiotropic associations between psychiatric disorders and autoimmune diseases have been reported [16], and so have sex-specific reverse genetic effects [36, 37]. Thus, we might hypothesize that the inverse associations observed in our study could be the result of pleiotropic variants, exhibiting sex-specific associations in opposite directions in either ADHD and/or IBD. Another potential mechanism could be that there is a tendency for more genetic variants positively associated with both ADHD and IBD to be located on the X-chromosome, while on the Y-chromosome, there is a greater burden of variants positively associated with ADHD, but negatively associated with IBD [35]. As the sex chromosomes have been largely ignored in GWASs owing to analytical difficulties, this is an area where further research is warranted.

Alternatively, smoking and BMI may play a role in the sex-discordant associations between ADHD and IBD [20, 47, 48, 52, 56]. However, adjustment for these potential mediators did not affect the associations much in our mother analyses, implying that they are weak mediators. Further, smoking has been shown to protect against UC, but confer risk for CD [51] and can consequently not easily explain our results as we then would have expected a negative association between ADHD and UC in females. Regarding BMI, prospective studies have only associated premorbid BMI with CD and not UC [56], which is not in agreement with our findings.

It could be that living with ADHD as a female gives rise to more stress, for example through social expectations and cultural norms, which again might lead to more autoimmunity [51, 65] and potentially, the sex-specific associations. However, one study showed that even though ADHD symptoms predispose to more stressful life events, female sex has been shown not to predispose to more stressful life events among those with ADHD symptoms [64].

Our study has several strengths. The use of compulsory population-wide registries minimize the risk of selection bias, and may provide the statistical power needed to investigate potential associations between ADHD and different autoimmune diseases. Further, the compulsory registration of prescription data protects against follow-up bias. However, we do not have information on medication given to hospital inpatients and nursing homes. Considering that the individuals in the primary analyses were all under 50 years, and that chronic diseases were investigated, we assume these factors to be of minor importance.

Another strength is the possibility to adjust for smoking and BMI in the mother analyses to assess mediating effects. However, we make the assumption that BMI and smoking at last registered pregnancy is “representative” of lifetime status up to 2015, which is sub-optimal. As the mother analyses were based on females, the generalizability to males could be questioned. Nonetheless, we consider it biologically unlikely that the positive association between psoriasis and ADHD is mediated purely by smoking and BMI in males, but not in females. In addition, several types of bias may occur as the mother analyses were based on only females who had given birth, and many autoimmune diseases are associated with reduced fertility [71], again possibly affecting the generalizability of the study. We are also aware that adjusting for intermediate variables, as we did in the mother analyses, may introduce collider stratification bias due to unmeasured variables affecting both smoking, BMI and the autoimmune diseases [57, 58]. Caution should therefore be exercised in the interpretation of these analyses. Also, we had problems with missing data for both smoking and BMI.

In Norway, the prescription of medication used in the treatment of ADHD is restricted and the drugs are only prescribed after thorough diagnostic evaluation in specialist health care. ADHD patients as defined by dispensed drugs is therefore presumably specific for ADHD. Nonetheless, we have missed patients who used ADHD medication only prior to 2004, and those who have never been prescribed medications due to contraindications, mild symptoms or patients who declined pharmacological treatment. However, a previous study using similar data from the same period, demonstrated that only 17% of registered ADHD patients had not received ADHD medication [24]. Furthermore, our ADHD case definition includes individuals who in 2004–2008 were prescribed stimulants for treatment of narcolepsy, but as this is a very minor number, it should not influence the results.

Dispensed medication and reimbursement codes (ICD-10 codes and ICPC codes as indications for dispensed medication) were used as proxies for autoimmune diseases. Thus, our definitions of autoimmune diseases may not capture all patients. For example, patients with primary-progressive multiple sclerosis, which constitute 10–15% of multiple sclerosis patients, will often not be identified by our approach as until recently there have been limited pharmacological treatment options for this group [72]. Further, the reimbursement codes may not always be used correctly. Despite the limitations of our disease identification, we believe that a drug prescribed with a reimbursement code, indicates thorough diagnostics, especially considering that many of the drugs may have serious side effects and are not used without due consideration.

We found no robust statistically significant associations between ADHD and the autoimmune diseases iridocyclitis, rheumatoid arthritis, SLE, ankylosing spondylitis, multiple sclerosis or type 1 diabetes. This could be due to a genuine lack of association between these autoimmune diseases and ADHD. Nonetheless, it could be that the low share of individuals born prior to 1990 who have dispensed ADHD-medication (supplementary Fig. 1) as compared to those born later, may reflect ADHD symptom remission before 2004 when the NorPD was established, or historical underdiagnosis and undertreatment of ADHD. Consequently, these ADHD individuals are not identified by our case definition. On the contrary, many autoimmune diseases are diagnosed later in life. Combined, our study may be inadequate for discovering associations between ADHD and autoimmune diseases with late debut. This may also partly explain the absence of any associations between ADHD and UC among individuals born 1985–2011 (supplementary material).

As our study is cross-sectional and we exclude all deceased or emigrated individuals, this may constitute a source of bias as ADHD is associated with increased mortality [73] and so are many of the autoimmune diseases [74, 75]. However, we do not believe that such bias underlies the findings of our study. First, the increased mortality associated with ADHD, mostly accidents, is unlikely to differ by autoimmune diseases nor constitute a large absolute number. Second, our cohort is relatively young with the oldest in the main analyses being 48 years of age at linkage. Therefore, most of the cohort is too young for cardiovascular death, which constitute a large portion of the mortality associated with autoimmune diseases [74–76]. In addition, the findings regarding CD and psoriasis could be identified among younger individuals in the birth year-stratified analyses. On the other hand, not excluding individuals who died or emigrated before the end of study could have lead to bias. Individuals who received ADHD medication after 2004, and thus captured by our study as ADHD patients, but developed autoimmune diseases and died or emigrated before 2008 would be lost.

Another source of bias could be that ADHD patients, already in contact with the health services, may get a diagnosis of comorbid diseases more easily than individuals who do not have an established link with the health services. However, one should then expect increased odds of all autoimmune diseases, and for both females and males, which was not the case. The symptoms of an autoimmune disease could also be mistaken for ADHD symptoms. For example the itch of psoriasis could lead to lower sleep quality and daytime sleepiness [77, 78], which may be mistaken for the impaired attention of ADHD. Yet, one would again expect increased odds of all autoimmune diseases.

In conclusion, our study supports previous reports on associations between ADHD and autoimmune diseases, and adds new knowledge about sex-specific associations and even reverse direction by sex for some associations. Our results also suggest that these associations are not mediated by smoking or BMI. Overall, our study suggests that sex-specific immune-mediated neurodevelopment may play a role in ADHD etiology, warranting further investigation. Future studies investigating the relationship between autoimmunity and neuropsychiatric disorders should be aware of sex-specific effects.

## Electronic supplementary material

Below is the link to the electronic supplementary material.

Supplementary material 1 (DOCX 44 kb)

**Supplementary Fig.** **1** Birth year and sex-specific prevalence rates for ADHD and the autoimmune diseases investigated in the primary analyses (PDF 16 kb)

**Supplementary Fig.** **2** Flowchart of inclusion in the mother analyses (PDF 23 kb)
