# Correction to: Associations between attention-deficit/hyperactivity disorder and autoimmune diseases are modified by sex: a population-based cross-sectional study

**DOI:** 10.1007/s00787-017-1087-7

**Published:** 2017-12-01

**Authors:** Tor-Arne Hegvik, Johanne Telnes Instanes, Jan Haavik, Kari Klungsøyr, Anders Engeland

**Affiliations:** 1 Department of Biomedicine, University of Bergen, Jonas Lies vei 91, N‑5009 Bergen, Norway; 2 K.G. Jebsen Centre for Neuropsychiatric Disorders, University of Bergen, Jonas Lies vei 91, N‑5009 Bergen, Norway; 3 Department of Global Public Health and Primary Care, University of Bergen, Bergen, Norway; 4 Division of Psychiatry, Haukeland University Hospital, Bergen, Norway; 5 Domain for Health Data and Digitalization, Norwegian Institute of Public Health, Bergen, Norway; 6 Department of Pharmacoepidemiology, Norwegian Institute of Public Health, Bergen/Oslo, Norway

## Correction to: Eur Child Adolesc Psychiatry 10.1007/s00787-017-1056-1

The article “Associations between attention-deficit/hyperactivity disorder and autoimmune diseases are modified by sex: a population-based cross-sectional study”, written by Tor-Arne Hegvik, Johanne Telnes Instanes, Jan Haavik, Kari Klungsøyr and Anders Engeland, was originally published electronically on the publisher’s internet portal (currently SpringerLink) on October 5, 2017 without open access due to an error by the Springer editorial office in the processing of this article. The authors had originally opted for open access.

The copyright of the article was therefore changed on November, 5 2017 to © The Author(s) 2017 and the article is forthwith distributed under the terms of the Creative Commons Attribution 4.0 International License (http://creativecommons.org/licenses/by/4.0/), which permits use, duplication, adaptation, distribution and reproduction in any medium or format, as long as you give appropriate credit to the original author(s) and the source, provide a link to the Creative Commons license, and indicate if changes were made.

The original article has been corrected.

