# Supplementary material for: Associations between attention-deficit/hyperactivity disorder and autoimmune diseases are modified by sex: a population-based cross-sectional study
Source: Eur Child Adolesc Psychiatry. 2017 Oct 5;27(5):663–75. doi: 10.1007/s00787-017-1056-1 (PMC5945751; doi:10.1007/s00787-017-1056-1)
Supplement: Supplementary file 1 — Supplementary material 1 (DOCX 44 kb) [file 787_2017_1056_MOESM1_ESM.docx]

**Associations between attention-deficit/hyperactivity disorder and autoimmune diseases are modified by sex: a population-based cross-sectional study**

Tor-Arne Hegvik1,2, Johanne Telnes Instanes1,2,3, Jan Haavik1,2,4, Kari Klungsøyr3,5, Anders Engeland3,6

1Department of Biomedicine, University of Bergen, Norway

2K.G. Jebsen Centre for Neuropsychiatric Disorders, University of Bergen, Norway

3Department of Global Public Health and Primary Care, University of Bergen, Norway

4Division of Psychiatry, Haukeland University Hospital, Bergen, Norway

5Domain for health data and digitalization, Norwegian Institute of Public Health, Bergen, Norway

6Department of Pharmacoepidemiology, Norwegian Institute of Public Health, Bergen/Oslo, Norway

E-mail address of corresponding author: Tor-Arne.Hegvik@uib.no

**Supplementary material**

**Psoriasis**

In the primary analysis, a robust association between attention-deficit/hyperactivity disorder (ADHD) and psoriasis was found. To confirm that this association was not due to an unknown bias affecting our case definition, we applied several stringent psoriasis case definitions. Psoriasis patients were first defined as those who had been dispensed the “topical antipsoriatics” calcipotriol (ATC D05AX02), calcitriol (ATC D05AX03) or calcipotriol combination (ATC D05AX52) which we believe to be specific, though not sensitive, for psoriasis (topical corticosteroids are commonly used in monotherapy) at least once. Secondly, as it is not unreasonable to believe psoriasis is more accurately diagnosed in specialist health care, psoriasis cases were defined as only those who had been prescribed a drug for psoriasis in specialist health care as defined by the ICD-10 code L40, effectively defining all those treated exclusively in general practice since 2008 as non-psoriasis individuals. Thirdly, psoriasis patients were defined as only those who had been dispensed two or more prescriptions for psoriasis based on ICD-10 L40 and/or ICPC S91. Similar analyses as in the primary analyses were then conducted.

All analyses confirmed the results of the primary analyses. See supplementary table 1.

**Supplementary table 1:** Associations between ADHD and psoriasis by different definitions among males and females, and the *p* value for the interaction between ADHD and sex, with adjustment for age and maternal education

|  | **Females** | | | **Males** | | | **All** |
| --- | --- | --- | --- | --- | --- | --- | --- |
| **Psoriasis case definition** | **n pr psoriasis case definition in total material** | **Adjusted for age n = 1 219 669 ADHD n = 22 878** | **Adjusted for age and maternal education**  **n = 1 207 694 ADHD n = 22 741** | **n pr psoriasis case definition in total material** | **Adjusted for age n = 1 280 449 ADHD n = 40 843** | **Adjusted for age and maternal education**  **n = 1 267 647 ADHD n = 40 544** | ***P* value of interaction between ADHD and sex (adjusted for age and maternal education) n = 2 475 341 ADHD n = 63 285** |
|  |  | OR (95% CI) | OR (95% CI) |  | OR (95% CI) | OR (95% CI) | *p* |
| Dispensed specific antipsoriatics^a^ | 18 173 | 1.51 (1.37-1.65) | 1.47 (1.34-1.62) | 18 557 | 1.33 (1.22-1.45) | 1.30 (1.20-1.42) | 0.0040 |
| Prescribed drug for psoriasis in specialist health care^b^ | 12 500 | 1.44 (1.28-1.61) | 1.42 (1.27-1.59) | 12 341 | 1.18 (1.06-1.31) | 1.17 (1.05-1.30) | 0.0014 |
| Dispensed a drug for psoriasis twice or more^c^ | 19 875 | 1.65 (1.51-1.80) | 1.61 (1.47-1.75) | 19 512 | 1.33 (1.22-1.44) | 1.30 (1.19-1.41) | 8.5 × 10^-7^ |
| ^a^ Calcipotriol (ATC D05AX02), calcitriol (ATC D05AX03) and calcipotriol combination (ATC D05AX52)  ^b^ Only based on reimbursement code ICD-code L40  ^c^ Based on reimbursement code ICD-10 L40 or ICPC S91 ADHD, attention-deficit/hyperactivity disorder; CI, confidence interval; OR, odds ratio | | | | | | | |

Further, we assessed whether there were any period effects on the association between psoriasis and ADHD. We grouped all birth years into 5-year categories and performed stratified analyses.

The results suggested that there were no major period effects. See supplementary table 2. For those born years 2002-2006, 9-13 years at time of linkage, there were no associations, for neither females nor males. However, for individuals born 2007-2011, 4-8 years at time of linkage, the association between ADHD and psoriasis was present for both sexes. The associations for 2007-2011 were primarily driven by prescriptions from general practitioners (ICPC) (not shown).

**Supplementary table 2:** Associations between ADHD and psoriasis per 5-year birth year category, 1967-2011

| **Females** |  |  |  |  |  |
| --- | --- | --- | --- | --- | --- |
| **Birth year** | **Total n** | **Psoriasis n** | **Psoriasis specialist n^a^** | **ADHD n** | **Non-adjusted** |
|  |  |  |  |  | OR (95% CI) |
| 1967-2011 | 1 219 669 | 32 190 | 12 500 | 22 878 | 1.47 (1.37-1.57) |
| 1967-1971 | 151 219 | 6937 | 2746 | 1643 | 1.67 (1.38-2.01) |
| 1972-1976 | 135 848 | 5819 | 2227 | 1784 | 1.65 (1.37-1.99) |
| 1977-1981 | 118 015 | 4478 | 1774 | 1804 | 1.32 (1.07-1.64) |
| 1982-1986 | 117 810 | 4235 | 1578 | 2261 | 1.35 (1.11-1.65) |
| 1987-1991 | 136 399 | 4081 | 1532 | 4129 | 1.61 (1.39-1.87) |
| 1992-1996 | 141 336 | 3176 | 1203 | 4770 | 1.29 (1.08-1.53) |
| 1997-2001 | 138 641 | 1963 | 804 | 3975 | 1.37 (1.09-1.73) |
| 2002-2006 | 135 761 | 1087 | 467 | 2225 | 1.24 (0.81-1.90) |
| 2007-2011 | 144 640 | 414 | 169 | 287 | 3.70 (1.18-11.59) |
| **Males** |  |  |  |  |  |
| **Birth year** | **Total n** | **Psoriasis n** | **Psoriasis specialist n^a^** | **ADHD n** | **Non-adjusted** |
|  |  |  |  |  | OR (95% CI) |
| 1967-2011 | 1 280 449 | 30 228 | 12 341 | 40 843 | 1.00 (0.94-1.07) |
| 1967-1971 | 156 295 | 7305 | 2992 | 1718 | 1.46 (1.20-1.76) |
| 1972-1976 | 140 673 | 6033 | 2545 | 2022 | 1.23 (1.01-1.50) |
| 1977-1981 | 123 417 | 4519 | 1919 | 2168 | 1.61 (1.34-1.94) |
| 1982-1986 | 124 579 | 3759 | 1565 | 2966 | 1.37 (1.14-1.65) |
| 1987-1991 | 143 863 | 3203 | 1220 | 6195 | 1.12 (0.95-1.32) |
| 1992-1996 | 150 394 | 2364 | 883 | 9364 | 1.23 (1.05-1.44) |
| 1997-2001 | 145 859 | 1562 | 611 | 9243 | 1.20 (0.99-1.45) |
| 2002-2006 | 142 557 | 955 | 398 | 6130 | 1.15 (0.86-1.55) |
| 2007-2011 | 152 812 | 528 | 208 | 1037 | 2.55 (1.32-4.95) |
| ^a^ Prescribed drug for psoriasis in specialist health care ADHD, attention-deficit/hyperactivity disorder; CI, confidence interval; OR, odds ratio | | | | | |

**Crohn’s disease and ulcerative colitis**

Sex-specific associations were noted between ADHD and Crohn’s disease (CD), and ADHD and ulcerative colitis (UC) in the primary analyses. However, as 2334 individuals had been defined as having both CD and UC (37.1% of the CD patients and 21.3% of the UC patients), we investigated whether these associations were linked to only one of the autoimmune diseases. To define “pure” CD and UC, all individuals with both diagnoses were redefined as having neither CD nor UC. Similar analyses to the primary analyses were then conducted.

The results confirmed the findings of the primary analysis for females, with ADHD being associated with increased risk of both CD, adjusted odds ratio (adjOR) = 1.52 (95%CI: 1.17-1.99), and UC, adjOR = 1.28 (95%CI: 1.03-1.58). However, the negative association between ADHD and CD among males did not pass the threshold for nominal statistical significance, adjOR = 0.79 (95%CI: 0.58-1.08) and no trend for association between ADHD and UC among males was noted. The interaction effects were still present, albeit attenuated. See supplementary table 3 for detailed results.

**Supplementary table 3:** Associations between ADHD and the autoimmune disorders Crohn’s disease and ulcerative colitis after redefining those diagnosed with both autoimmune diseases as having neither

| **Autoimmune disease** | **Females** | | | **Males** | | | **All** |
| --- | --- | --- | --- | --- | --- | --- | --- |
|  | **n** | **Adjusted for age n = 1 219 669 ADHD n =22 878** | **Adjusted for age and maternal education**  **n = 1 207 694 ADHD n = 22 741** | **n** | **Adjusted for age n = 1 280 449 ADHD n = 40 843** | **Adjusted for age and maternal education**  **n = 1 267 647 ADHD n = 40 544** | ***P* value of interaction between ADHD and sex (adjusted for age and maternal education) n = 2 475 341 ADHD n = 63 285** |
|  |  | OR (95% CI) | OR (95% CI) |  | OR (95% CI) | OR (95% CI) | *p* |
| Crohn’s disease | 2075 | 1.56 (1.20-2.04) | 1.52 (1.17-1.99) | 1883 | 0.80 (0.58-1.09) | 0.79 (0.58-1.08) | 0.0021 |
| Ulcerative colitis | 4183 | 1.27 (1.02-1.57) | 1.28 (1.03-1.58) | 4443 | 0.95 (0.78-1.16) | 0.94 (0.77-1.15) | 0.046 |
| ADHD, attention-deficit/hyperactivity disorder; CI, confidence interval; OR, odds ratio | | | | | | | |

Period and age-effects may play a role in the associations between ADHD and the inflammatory bowel disorders. We therefore stratified the sample into those born in 1985 or earlier, 30 years or older at linkage, and those born after 1985, 29 or younger at linkage. Logistic regression analyses similar to the main analyses were then conducted stratified on the age groups, including adjusting for age as a linear covariate and maternal education.

For CD, the results were largely in line with the main analyses, if to accept non-statistically significant trends. In females, ADHD increased the odds of CD, while male sex lowered the odds. Age and period effects were of minor importance. However, for UC, only among those born 1967-1985, were the results similar to the main analyses, with ADHD conferring increased odds of UC among females, and decreased odds among males. For individuals born 1986-2011, no association between ADHD and UC was noted in either sex. See supplementary table 4 for detailed results.

**Supplementary table 4:** Associations between ADHD and the autoimmune diseases Crohn’s disease and ulcerative colitis after stratification on born in or before 1985, or after 1985.

| **Autoimmune disease** | **Females born 1967-1985** | | **Females born 1986-2011** | | **Males born 1967-1985** | | **Males born 1986-2011** | | **All born**  **1967-1985** | **All born 1986-2011** |
| --- | --- | --- | --- | --- | --- | --- | --- | --- | --- | --- |
|  | **Adjusted for age and maternal education**  **n = 497 034 ADHD n = 6942** | | **Adjusted for age and maternal education**  **n = 710 660 ADHD n = 15 799** | | **Adjusted for age and maternal education**  **n = 517 374 ADHD n = 8114** | | **Adjusted for age and maternal education**  **n = 750 273 ADHD n = 32 430** | | ***P* value of interaction between ADHD and sex (adjusted for age and maternal education) n = 1 014 408 ADHD n = 15 056** | ***P* value of interaction between ADHD and sex (adjusted for age and maternal education) n = 1 460 933 ADHD n = 48 229** |
|  | n | OR (95% CI) | n | OR (95% CI) | n | OR (95% CI) | n | OR (95% CI) | *p* | *p* |
| Crohn’s disease | 1946 | 1.36 (0.98-1.88) | 1321 | 1.25 (0.93-1.68) | 1711 | 0.66 (0.42-1.05) | 1281 | 0.65 (0.47-0.89) | 0.0095 | 0.0025 |
| Ulcerative colitis | 3638 | 1.32 (1.04-1.69) | 1737 | 1.01 (0.76-1.34) | 3773 | 0.67 (0.49-0.92) | 1772 | 0.93 (0.74-1.17) | 7.2 × 10^-4^ | 0.68 |
| ADHD, attention-deficit/hyperactivity disorder; CI, confidence interval; OR, odds ratio | | | | | | | | | | |
