# Supplementary material for: Associations between attention-deficit/hyperactivity disorder and autoimmune diseases are modified by sex: a population-based cross-sectional study
Source: Eur Child Adolesc Psychiatry. 2017 Oct 5;27(5):663–75. doi: 10.1007/s00787-017-1056-1 (PMC5945751; doi:10.1007/s00787-017-1056-1)
Supplement: Supplementary file 2 — Supplementary Fig. 1 Birth year and sex-specific prevalence rates for ADHD and the autoimmune diseases investigated in the primary analyses (PDF 16 kb) [file 787_2017_1056_MOESM2_ESM.pdf]

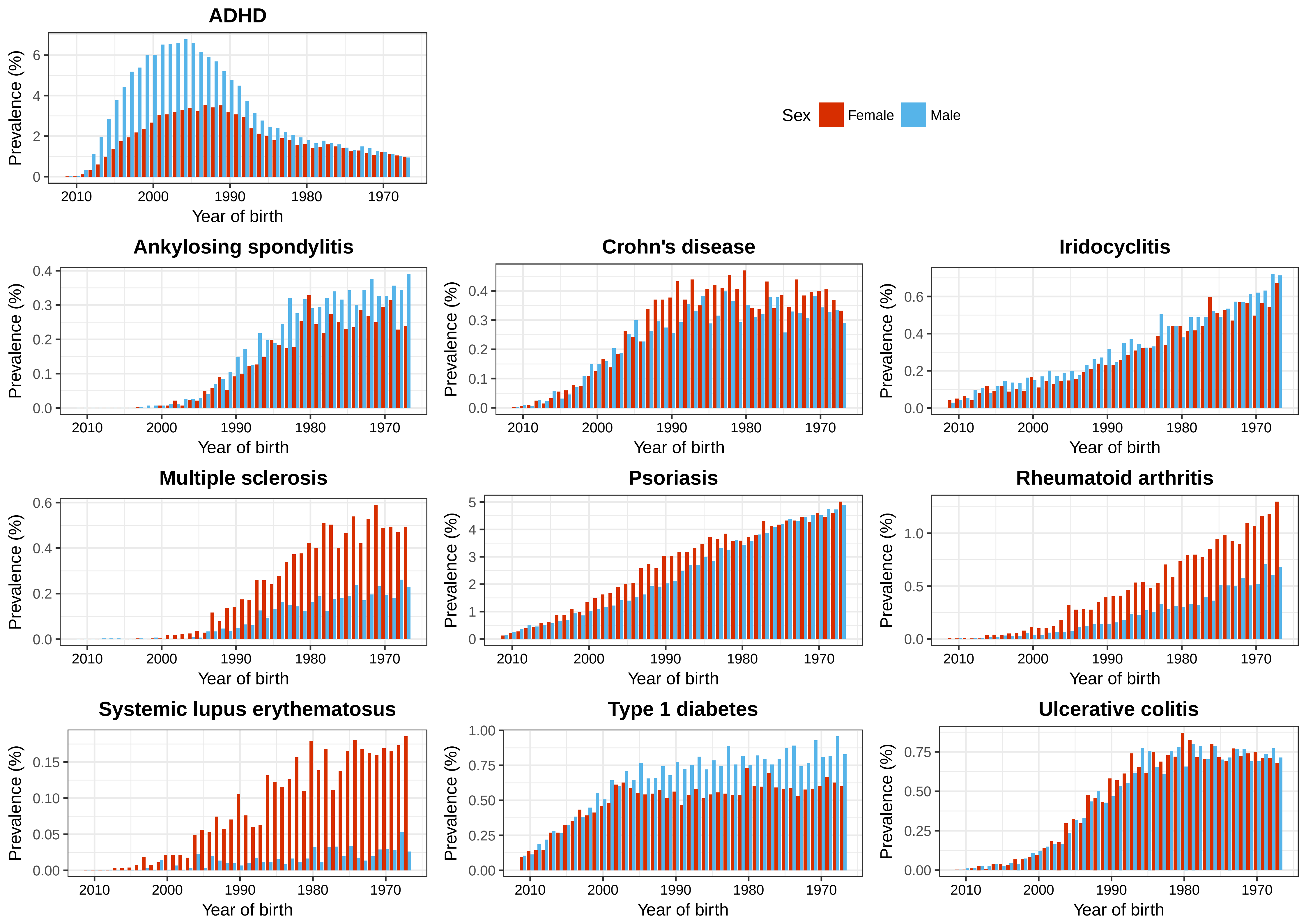

ADHD
Ankylosing spondylitis
Crohn's disease
Iridocyclitis
Multiple sclerosis
Psoriasis
Rheumatoid arthritis
Systemic lupus erythematosus
Type 1 diabetes
Ulcerative colitis
Prevalence (%)
Year of birth
Sex
Female
Male
