# Supplementary material for: Associations between attention-deficit/hyperactivity disorder and autoimmune diseases are modified by sex: a population-based cross-sectional study
Source: Eur Child Adolesc Psychiatry. 2017 Oct 5;27(5):663–75. doi: 10.1007/s00787-017-1056-1 (PMC5945751; doi:10.1007/s00787-017-1056-1)
Supplement: Supplementary file 3 — Supplementary Fig. 2 Flowchart of inclusion in the mother analyses (PDF 23 kb) [file 787_2017_1056_MOESM3_ESM.pdf]

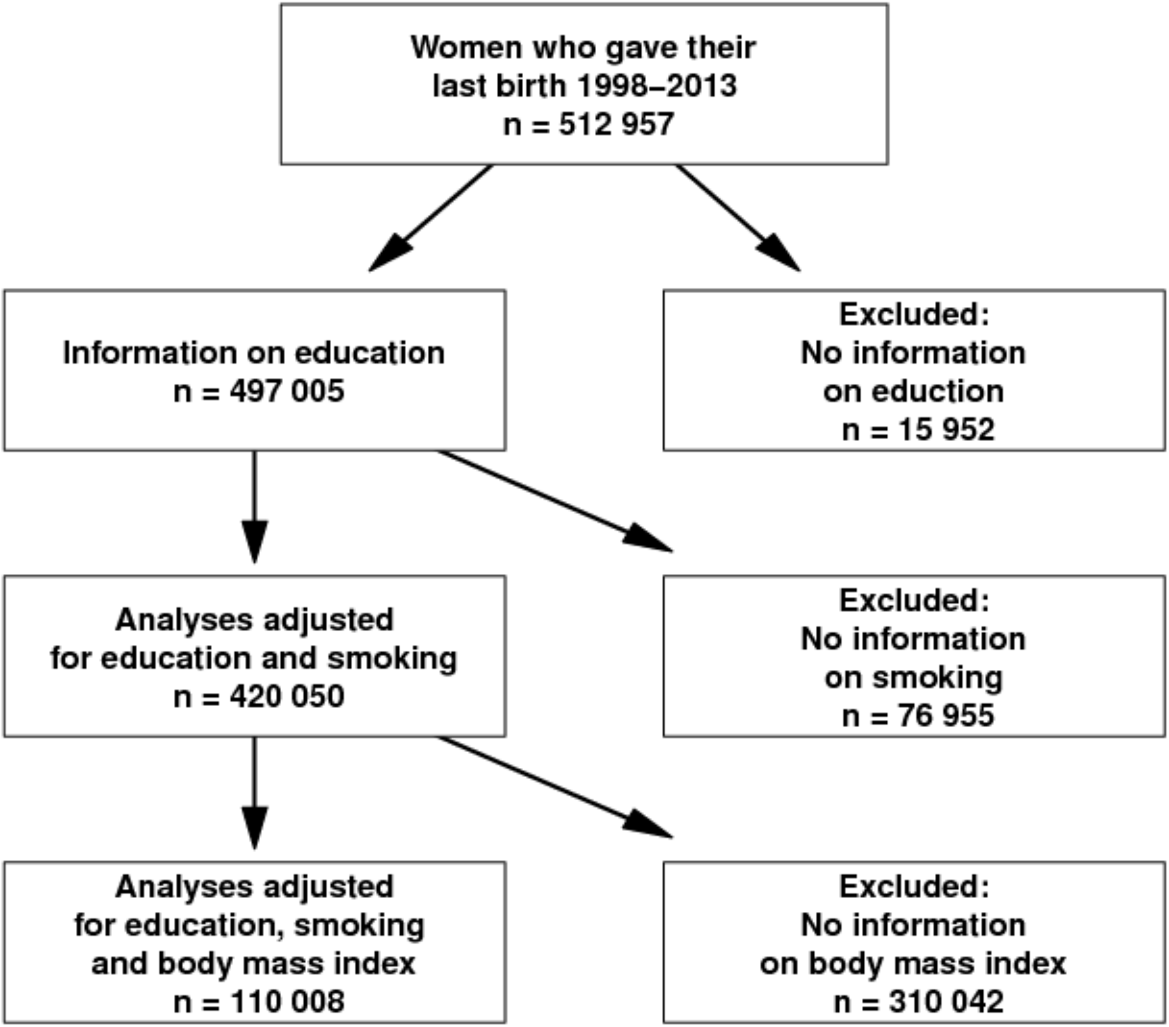
Women who gave their last birth 1998–2013 n = 512 957
Information on education n = 497 005
Excluded: No information on eduction n = 15 952
Analyses adjusted for education and smoking n = 420 050
Excluded: No information on smoking n = 76 955
Analyses adjusted for education, smoking and body mass index n = 110 008
Excluded: No information on body mass index n = 310 042
